# Molecular inimitability amongst tumors: implications for precision cancer medicine in the age of personalized oncology

**DOI:** 10.18632/oncotarget.5289

**Published:** 2015-09-16

**Authors:** Sandip P. Patel, Maria Schwaederle, Gregory A. Daniels, Paul T. Fanta, Richard B. Schwab, Kelly A. Shimabukuro, Santosh Kesari, David E. Piccioni, Lyudmila A. Bazhenova, Teresa L. Helsten, Scott M. Lippman, Barbara A. Parker, Razelle Kurzrock

**Affiliations:** ^1^ Center for Personalized Cancer Therapy and Division of Hematology and Oncology, University of California San Diego Moores Cancer Center, La Jolla, CA, USA

**Keywords:** personalized medicine, genomics, cancer, next-generation sequencing, clinical trials

## Abstract

Tumor sequencing has revolutionized oncology, allowing for detailed interrogation of the molecular underpinnings of cancer at an individual level. With this additional insight, it is increasingly apparent that not only do tumors vary within a sample (tumor heterogeneity), but also that each patient's individual tumor is a constellation of unique molecular aberrations that will require an equally unique personalized therapeutic regimen. We report here the results of 439 patients who underwent Clinical Laboratory Improvement Amendment (CLIA)-certified next generation sequencing (NGS) across histologies. Among these patients, 98.4% had a unique molecular profile, and aside from three primary brain tumor patients with a single genetic lesion (*IDH1 R132H*), no two patients within a given histology were molecularly identical. Additionally, two sets of patients had identical profiles consisting of two mutations in common and no other anomalies. However, these profiles did not segregate by histology (lung adenocarcinoma-appendiceal cancer (*KRAS G12D* and *GNAS R201C)*, and lung adenocarcinoma-liposarcoma (*CDK4* and *MDM2* amplification pairs)). These findings suggest that most advanced tumors are molecular singletons within and between histologies, and that tumors that differ in histology may still nonetheless exhibit identical molecular portraits, albeit rarely.

## INTRODUCTION

The advent of molecular-guided precision medicine is impacting cancer therapy with an array of impressive responses in patients who undergo matched targeted therapy. Multidisciplinary molecular tumor boards are beginning to help navigate this complex mutational landscape [1, 2]. The utilization of novel targeted therapies such as imatinib in chronic myelogenous leukemia and trastuzumab in HER2+ breast cancer, have transformed the field [3, 4]. More recently, a myriad of other targeted agents, such as erlotinib in *EGFR*-mutated non-small cell lung cancer and vemurafenib or dabrafenib in *BRAF*-mutated melanoma among others, have shown success [5, 6, 7].

Traditionally, molecular testing for a relatively small panel of genes was conducted in a histologically-defined subset of patients to determine therapy (e.g. *BRAF* mutation testing in patients with melanoma, HER2 immunohistochemical staining in patients with breast cancer). However, with the advent of multiplex methods such as next-generation sequencing (NGS), detection of similar mutations in other tumor types led to the utilization of targeted therapy outside of its initial histologic classification. For example, BRAF inhibitors have shown promising efficacy in *BRAF*-mutated lung and thyroid cancers as well as in hairy cell leukemia, indicating that oncogenic driver mutations do not necessarily segregate by histology, and matched molecular therapy is effective across many, but not all, histologies [8, 9, 10, 11, 12].

Beyond identification of the affected gene, the precise genetic aberration has important clinical implications. For example, in *EGFR*-mutated non-small cell lung cancer (NSCLC), only individuals with activating mutations in exons 19 and 21 (L858R) are sensitive to the first-generation EGFR inhibitors gefitinib, erlotinib, and afatinib while those with exon 20 T790M mutations or in-frame insertions and/or duplications (codons 767 to 774) are resistant to these agents [13, 14, 15]. Patients treated with imatinib for gastrointestinal stromal tumor (GIST) with c-KIT mutations in exon 11 have an 83.5% response rate versus 47.8% in patients with exon 9 mutations [16], and *PIK3CA* H1047R mutations may be more sensitive to PI3K/Akt/mTor inhibitors than other *PIK3CA* mutations [11]. Thus, understanding the underlying genetic defect in cancers requires identification of the culprit gene, as well as the particular alteration in that gene, in order to better predict responses to targeted therapy [15].

Based on CLIA-certified NGS of 439 tumors at UC San Diego Moores Cancer Center, we report here that 432 (98.4%) patients had a unique molecular profile. The seven individuals who were not molecular singletons include three with primary brain tumors and a solitary molecular lesion (*IDH1* R132H mutation), and two sets of tumor molecular twins that had two alterations in common—however, these alterations did not segregate by histology (lung adenocarcinoma-appendiceal cancer (*KRAS G12D* and *GNAS R201C*), and lung adenocarcinoma-liposarcoma (*CDK4* and *MDM2* amplification pairs)). These findings demonstrate that the vast majority of cancer patients have molecularly unique tumors, and that patients may be more similar to others with a different histology than to patients with the same histology. This finding that has important implications for defining cancer nosology, clinical trial design and regulatory approval of new agents.

## RESULTS

### Alterations per patient

**Figure 1:**
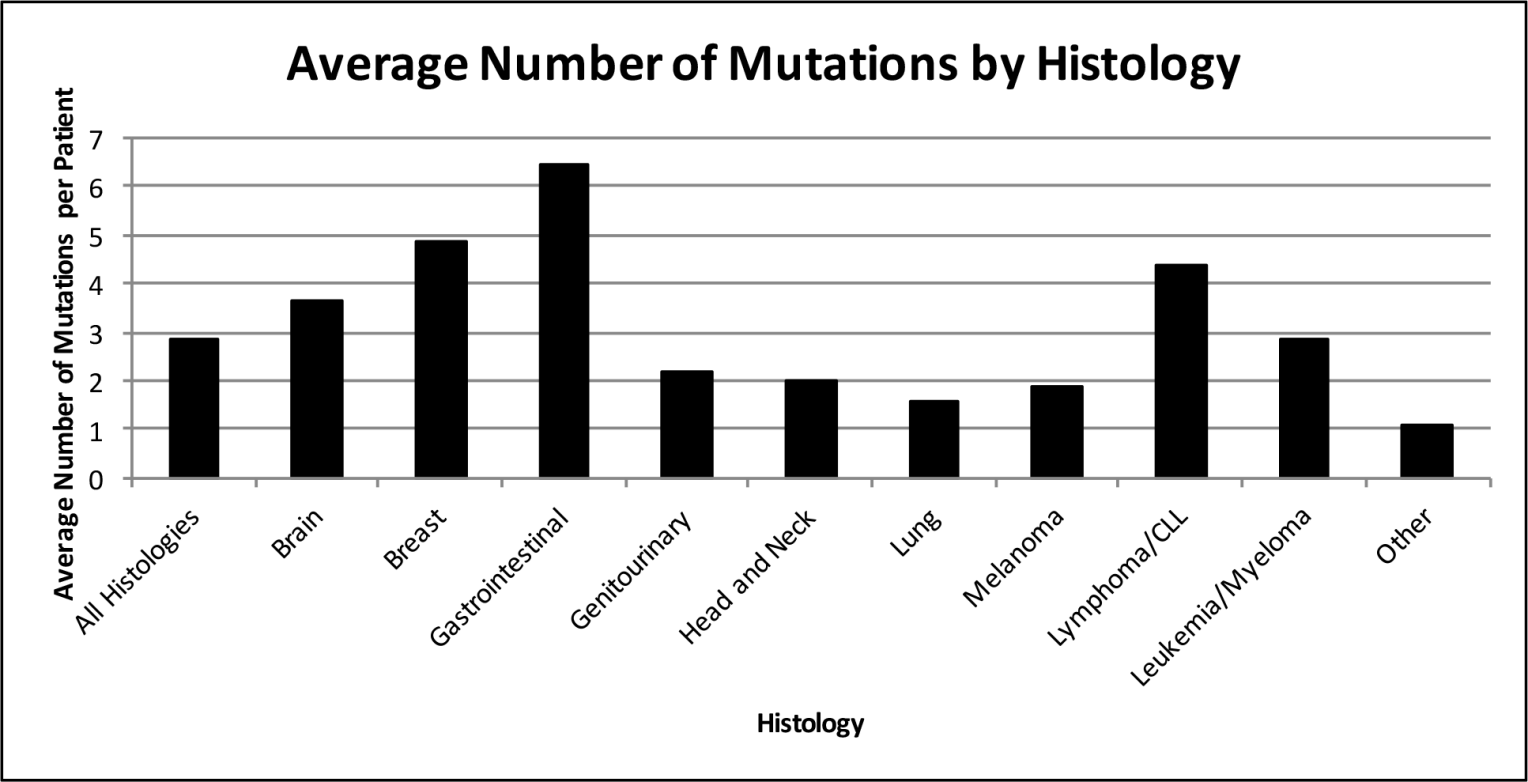
Average number of molecular aberrations by histology (*N* = 439 patients) Average number of molecular aberrations detected per patient by histology. Gastrointestinal cancers, breast cancer, and lymphoma had the highest number of mutations per tumor sample. Lung, head and neck, and genitourinary cancers had the fewest number of mutations as a histologic group.

**Figure 2:**
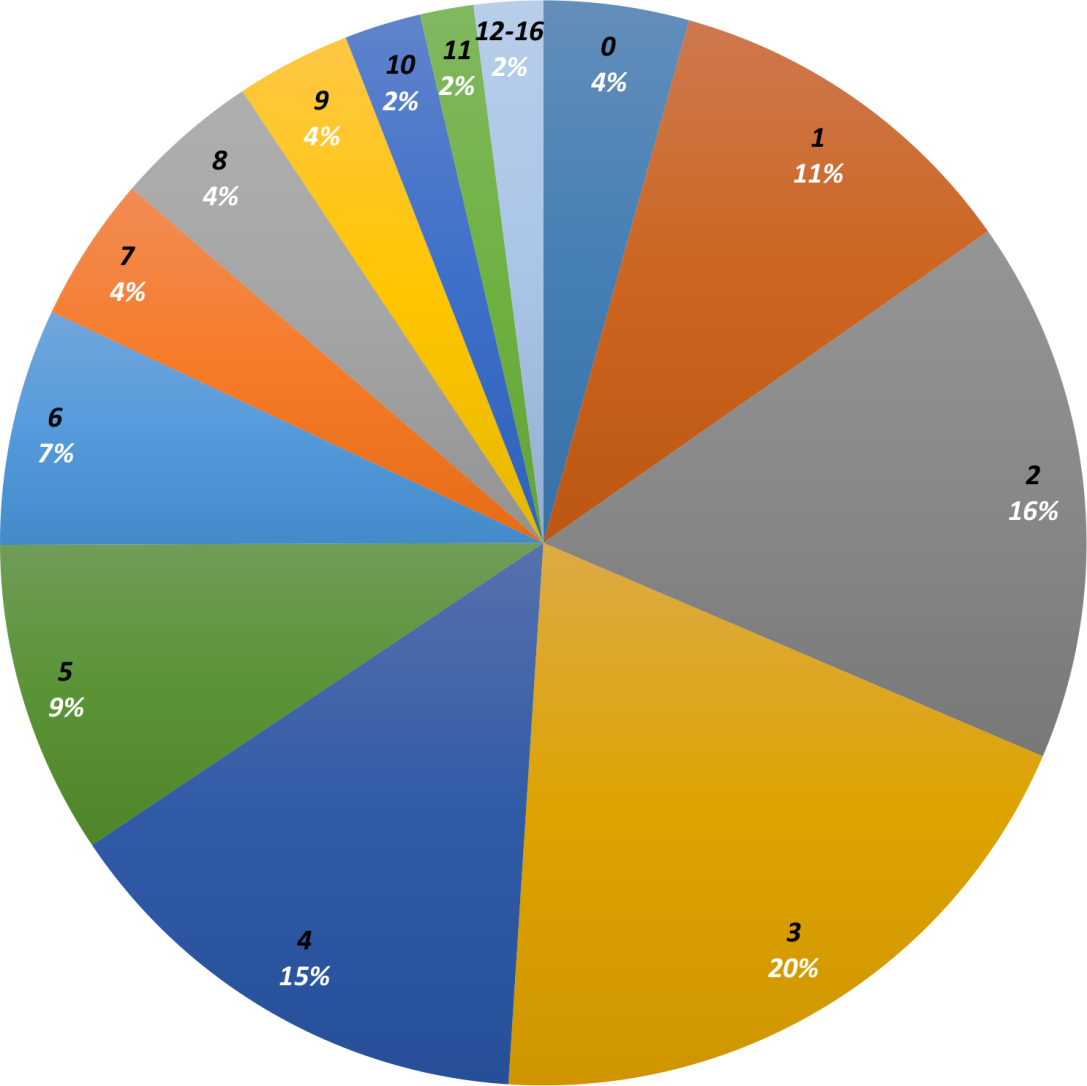
Distribution of number of alterations per patient Distribution of number of mutations (in black, increasing number of mutations in clockwise direction) detected in patient tumor specimens (number in white represents percentage of patients with number of mutations in black). A plurality of patients (20%) had 3 mutations detected on tumor sequencing. No mutations were detected in 4% of patients, while 12–16 mutations were detected in 2% of patients.

**Figure 3:**
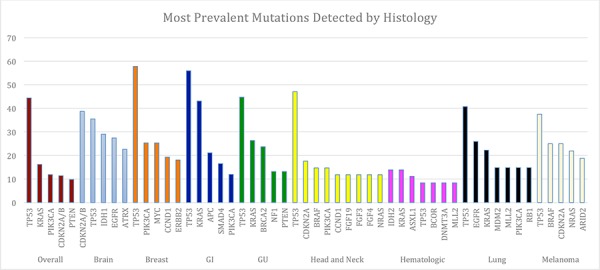
Most Common Genes Altered Overall and by Histology Five most commonly altered genes overall (brown) and by disease type with percent tumor samples with that mutation. *TP53* was the most common mutation detected overall and across all histologic subgroups except for in primary CNS tumors (CDKN2A and CDKN2B abnormalities more common) and in the hematologic malignancies (IDH2, KRAS, and ASXL1 more common). For some tumor types more than five genes are displayed, as frequency was the same for these genes.

Only 19 patients (4%) had no detectable genomic alterations. This group consisted of five patients with malignant hematologic conditions, three primary central nervous system (CNS) tumors, four gastrointestinal (GI) tumors, two cutaneous primary, two head and neck tumors, one breast tumor, and two cancers of unknown primary. Patients with gastrointestinal malignancies had the highest number of mutations on average (*N* = 6.5) (Figure 1). Missense mutations were the most common aberrations detected (32.5% of aberrations) followed by amplifications (28.7% of aberrations). The median number of alterations per patient was three, ranging from zero (19 patients, 4.3%) to 16 (1 patient with colorectal cancer, 0.2%) (Figure 2). The most common number of alterations (mode) detected was three (86 patients, 19.6%). The most common mutations detected were in *TP53*, present in 195 patients (44%), followed by *KRAS*, which were detected in 71 patients (16%) (Figure 3). Molecular testing was performed on primary tumor specimens in 58.1% of cases, and on metastatic lesions in 36.2% of cases. No matched pairs of concurrent primary and metastatic tumors were tested in this analysis. Between primary and metastatic lesions (unmatched), there was no difference in the number of alterations (mean of 4.1 alterations for primary site vs 4.4 for metastatic sites; *p* = 0.311). Additionally, the prevalence of common mutations listed above did not vary significantly between primary and metastatic site.

### Uniqueness of the molecular portfolios

Of 439 patients who underwent CLIA-certified next-generation sequencing, 432 (98.4%) had a unique molecular profile (Table 1). Aside from three primary central nervous system (CNS) tumors (2 oligodendrogliomas and 1 glioblastoma) with a single genetic lesion (*IDH1* R132H mutation), no two patients within a given histology or organ-based diagnosis were molecularly identical (Table 2). Additionally, two sets of patients had tumors that were molecular identical twins, but these portfolios did not segregate by organ-based diagnosis: *KRAS* G12D and *GNAS* R201C without any other alterations were both found in one patient with each of lung adenocarcinoma and appendiceal cancer; *CDK4* and *MDM2* amplifications were found in one patient with lung adenocarcinoma and one with liposarcoma.

**Table 1:** Demographic Data*

**Age**	
Median Age	54.3 years
Age < 60 years	63.5%
Age ≥ 60 years	36.5%
**Gender**	
Men	44%
Women	56%
**Ethnicity**	
African-American	2.7%
Asian	6.6%
Caucasian	72.9%
Hispanic	1.4%
Native American	0.5%
Other	13.4%
Unknown	2.5%
**Histology**	
Brain	14.1%
Breast	18.9%
Gastrointestinal	24.8%
Genitourinary	8.7%
Head and Neck	7.8%
Lung	6.2%
Melanoma	7.3%
Malignant Hematologic Condition	8.2%
Other	4.2%
**Matches**	
Genomic Match	11.2%
Molecular Match	1.6%
**Biopsy Site for Molecular Studies**	
Primary Tumor Site	58.1%
Metastatic Site	36.2%
Unknown Site	5.7%

* Characteristics of patients and tumor samples analyzed for this study. Brain includes primary CNS tumors only. Genomic match refers to gene-level matching between patient tumor specimens. Molecular match refers to both gene-level and allele-level matching between patient tumor specimens. Gastrointestinal and breast cancers were the most frequently profiled histologic subtypes, and the primary tumor site was most often sent for molecular testing.

**Table 2:** Molecular Twins*

Complete Molecular Profile	Histology 1	Histology 2	Histology 3
*IDH1 R132H*	Oligodendroglioma	Oligodendroglioma	Glioblastoma
*KRAS G12D and GNAS R201C*	Lung adenocarcinoma	Appendiceal carcinoma	—
*CDK4 and MDM2 amplification*	Liposarcoma	Lung adenocarcinoma	—

* Only the patients shown above had identical molecular profiles (molecular twins). IDH1 R132H was found in three primary CNS tumors. KRAS G12D with GNAS R201C was found in one patient with lung adenocarcinoma as well as in a patient with appendiceal carcinoma. CDK4 amplification and MDM2 amplification were found in liposarcoma and lung adenocarcinoma.

Examining patients at the genomic level, there were 49 of the 439 individuals (11.2%) studied, who had the same genes involved (albeit with differing alterations within these genes). These included the seven patients mentioned above that each had at least one molecular twin amongst them, as well as 42 tumors amongst which each patient had at least one gene level twin. Examining the 42 patients mentioned above, 26 patients had at least one gene-level twin amongst them with the same histology; 16 patients had at least one different-histology gene level twin amongst them. Within histologies, the only molecularly identical patients were the three patients with primary CNS glial tumors with solitary *IDH1* R132H mutation (Table 2).

## DISCUSSION

The discovery and clinical benefit of targeting oncogenic drivers such as *ALK, BCR-ABL, BRAF, EGFR*, and *HER2*, among others, has revolutionized medical oncology and ushered in the era of personalized/precision medicine. Increasingly sophisticated methods of tumor genomic analysis, including NGS, have allowed for an improved understanding of the molecular landscape of tumors with an exponential decline in costs [18]. As multiplexing technologies have improved to allow greater numbers of genes to be interrogated with lower tissue requirements, an increased genetic diversity has been unmasked—not only between patients, but within each tumor [19]. The Pan-Cancer analysis project from The Cancer Genome Atlas (TCGA) has analyzed molecular aberrations, initially amongst 12 histologies, and has determined 17 mutational signatures across histologies in addition to 14 copy number signatures, with *TP53* aberrations common across both signatures [20]. In our dataset, mutations in *TP53* were the most commonly detected aberration (44% of patients) followed by *KRAS* mutations (16%).

In our experience, 98.4% of patients had a unique molecular profile by targeted NGS of tumor. This finding has major implications for clinical trial design and the regulatory framework for drug approval. As an increasing number of rationally designed, targeted cancer therapeutics are developed, the number of patients in the potential study population with that particular aberration decreases. However, the potential for response in that smaller biomarker-selected population increases proportionally, resulting in an asymptotic predilection for personalized “n of one” trials to maximize clinical efficacy. For example, the pivotal trial for FDA approval of pemetrexed in non-squamous, non-small cell lung cancer (NSCLC) was a randomized study with 1,725 patients and demonstrated an overall survival benefit of 1.7 months compared to standard chemotherapy [21]. In contrast, the FDA approval of ceritinib was based on a single-arm phase 1 trial with 163 patients who had progressed and were intolerant to crizotinib [22]. The primary endpoint supporting approval was an objective response rate of 44% (95% CI 36%-52%) and duration of response of 7.1 months. Though these results indicate substantial improvement, further lengthening of response duration may require combinations of drugs customized to individual molecular portfolios. With the observation that molecular portfolios within and between cancers are mostly distinct, and that the small number of patients with tumors that are molecular twins are just as likely to have distinct—rather than similar, histologies—it becomes clearer that new approaches are needed. These may include efforts to understand convergent pathways, or, alternatively, entirely novel paradigms where the strategy of molecular matching is tested, while the actual drugs given to each patient differ [23]. A broad genomic approach to next-generation sequencing may be advantageous as the rapid development of novel therapeutics and an improved understanding of pathway signaling increase the number of potential efficacious therapeutic options [24]. Moreover, biomarkers for immune checkpoint blockade, one of the most promising areas of oncology, will require whole exome sequencing for neoantigen prediction and the development of personalized immunotherapeutic biomarkers [25].

A limitation of our study is the lack of whole exome sequencing or interrogation of the transcriptome or proteome; however to date “omics” beyond targeted NGS has usually not been performed as part of routine clinical testing or as part of broad-based genomically-driven cooperative group trials such as NCI-MATCH [26]. However, integrated “omics” platforms are likely to detect additional alterations and may demonstrate increased molecular diversity beyond what is reported here. Other limitations include the relatively small numbers of patients within some histologies.

Overall, our study shows that the rate of genomic twins (patients who have tumors with an identical gene-level aberration) was only 11.2%. The rate of molecular twins (tumors in which the genes affected and the precise site and type of aberration within the gene was identical) was even lower—1.6%. It is plausible that earlier in the disease course, there might be fewer aberrations and more homogeneity [27]. Furthermore, molecular abnormalities mostly did not segregate by tumor histology, a finding consistent with other studies [28, 29]. Therapies such as trastuzumab work against HER2-overexpressing tumors regardless of whether they originate in breast or gastric tissue, and clinical trials (NCT02091141, NCT01833169, NCT01831726, NCT01885195, NCT01981187 and NCT02002689) are testing this approach in a histology-agnostic manner and whether “oncogenic fraternal twins” can be treated effectively as such [4, 30, 31]. While molecular heterogeneity within breast cancer patients has been reported, our study broadens the histologic heterogeneity of tumor molecular profiling to include other solid tumors as well as primary CNS tumors and malignant hematologic conditions [32, 33]. Importantly, with our increased knowledge of oncogenic pathways and an expanding pipeline of targeted agents, the vast majority of patients (~90%) now have at least one potentially actionable aberration [34, 35]. As technology improves and biomarkers become more defined, the promise of precision cancer medicine with a personalized approach for each patient is becoming a necessity. The advent of immunotherapy has led to the emergence of additional predictive biomarkers, including mutational burden at a histologic level and PD-L1 immunohistochemistry or MSI-H at an individual level [36, 37, 38]. Patients who receive molecularly matched therapy, regardless of histology, have improved responses to therapy and longer survival [2, 12, 39]. Ultimately, novel clinical trial designs that deploy advanced genomics and other methodologies to interrogate tumors, and the utilization of rationally-designed customized therapeutic combinations early in the treatment course to prosecute tumors will be required in order to better serve patients with cancer.

## MATERIALS AND METHODS

Four hundred and thirty-nine patients seen at UC San Diego Moores Cancer Center with advanced malignancies who underwent CLIA)-certified next generation sequencing (182 or 236 genes analyzed via FoundationOne, Cambridge MA) starting in December 2012 had their tests results and clinical data recorded. All data was extracted by M.S. from the electronic medical record. This study was performed and consents obtained in accordance with UCSD Institutional Review Board guidelines

Genomic alterations detected included base substitutions, insertions, deletions, and copy number alterations [17]. DNA was extracted from 40 μm of formalin-fixed paraffin-embedded tissue (minimum 20% tumor cells) using the Maxwell 16 FFPE Plus LEV DNA Purification kit (Promega) and quantified using a standardized PicoGreen fluorescence assay (Invitrogen). Library construction was performed using 50–200ng of DNA sheared by sonication to approximately 100–400 bp before end-repair, dA addition and ligation of indexed, Illumina sequencing adaptors. Enrichment of target sequences (all coding exons of 182 or 236 cancer-related genes and selected introns from 28 genes recurrently rearranged in cancer) was achieved by solution-based hybrid capture with custom biotinylated oligonucleotide baits. Enriched libraries were sequenced to an average median depth of > 500X with 99% of bases covered > 100X (Illumina HiSeq 2000 platform using 49 × 49 paired-end reads) and mapped to the reference human genome (hg19) using the Burrows-Wheeler Aligner and the publicly available SAMtools, Picard, and Genome Analysis Toolkit. Point mutations were identified by a Bayesian algorithm; short insertions and deletions, determined by local assembly; gene copy number alterations (amplifications), by comparison to process matched normal controls; and gene fusions/rearrangements, by clustering chimeric reads mapped to targeted introns. Genes were considered amplified if at eight-fold copy number except for *ERBB2*, which is amplified at seven-fold copy number.

### Molecular profiles and definitions

If two or more patients had involvement of the same altered genes, but the exact locus in the genes differed, they were considered identical at the gene, but not at the molecular level (termed genomic but not molecular twins). Tumors were defined as having a unique molecular and genomic portfolio if they were non-identical in both their molecular and their genomic abnormalities (termed molecularly unique).
